# Pyridinium [2,6-bis­(5-phenyl-1*H*-pyrazol-3-yl-κ*N* ^2^)pyridine-κ*N*]tetra­nitrato-κ^6^ *O*,*O*′;κ*O*-samarium(III) pyridine monosolvate

**DOI:** 10.1107/S1600536811022653

**Published:** 2011-06-18

**Authors:** Shui Hu, Yongfeng Zhao, Huai-Ming Hu, Li Liu

**Affiliations:** a Key Laboratory of Carbon Fiber and Functional Polymers, Ministry of Education, Beijing University of Chemical Technology, Beijing 100029, People’s Republic of China; b College of Chemistry and Materials Science, Northwest University, Xi’an 710069, People’s Republic of China

## Abstract

In the title compound, (C_5_H_6_N)[Sm(NO_3_)_4_(C_23_H_17_N_5_)]·C_5_H_5_N, the Sm^III^ atom is ten-coordinated by the *N*,*N*′,*N*′′-tridentate bis­(pyrazole) ligand and seven O atoms from four nitrate anions (three bidentate and one monodentate). The dihedral angles between the central pyridine ring and adjacent pyrazole rings in the ligand are 1.3 (2) and 3.2 (2)°; the dihedral angles between the pyrazole rings and their pendant phenyl rings are 42.0 (3) and 16.1 (2)°. The conformation of the anionic complex ion is supported by an intra­molecular N—H⋯O hydrogen bond. In the crystal, inversion dimers linked by pairs of N—H⋯O hydrogen bonds occur. The pyridinium cation forms an N—H⋯N hydrogen bond.

## Related literature

For the synthesis of the ligand, see: Zhao *et al.* (2009 ▶). For related transition metal and lanthanide complexes, see: Argent *et al.* (2005 ▶); Bardwell *et al.* (1997 ▶); Barrios *et al.* (2008 ▶); Coronado *et al.* (2003 ▶); Dong *et al.* (1999 ▶); Dutta *et al.* (1996 ▶); Gamez *et al.* (2002 ▶); Gimenez-Lopez *et al.* (2005 ▶); Scudder *et al.* (2005 ▶); Wei *et al.* (2008 ▶).
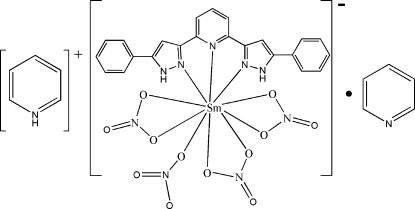


## Experimental

### 

#### Crystal data

(C_5_H_6_N)[Sm(NO_3_)_4_(C_23_H_17_N_5_)]·C_5_H_5_N
*M* *_r_* = 921.01
Triclinic, 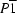

*a* = 10.5234 (19) Å
*b* = 12.826 (2) Å
*c* = 14.190 (3) Å
α = 75.970 (2)°
β = 86.453 (2)°
γ = 84.108 (2)°
*V* = 1847.0 (6) Å^3^
*Z* = 2
Mo *K*α radiation
μ = 1.67 mm^−1^
*T* = 296 K
0.18 × 0.12 × 0.10 mm

#### Data collection

Bruker APEXII CCD diffractometer
Absorption correction: multi-scan (*SADABS*; Bruker, 2005 ▶) *T*_min_ = 0.753, *T*_max_ = 0.851
9121 measured reflections
6391 independent reflections
5442 reflections with *I* > 2σ(*I*)
*R*_int_ = 0.020

#### Refinement

*R*[*F* ^2^ > 2σ(*F* ^2^)] = 0.035
*wR*(*F* ^2^) = 0.086
*S* = 1.09
6391 reflections
517 parameters
1 restraint
H atoms treated by a mixture of independent and constrained refinement
Δρ_max_ = 0.95 e Å^−3^
Δρ_min_ = −0.67 e Å^−3^

### 

Data collection: *APEX2* (Bruker, 2005 ▶); cell refinement: *SAINT* (Bruker, 2005 ▶); data reduction: *SAINT*; program(s) used to solve structure: *SHELXS97* (Sheldrick, 2008 ▶); program(s) used to refine structure: *SHELXL97* (Sheldrick, 2008 ▶); molecular graphics: *SHELXTL* (Sheldrick, 2008 ▶); software used to prepare material for publication: *SHELXTL*.

## Supplementary Material

Crystal structure: contains datablock(s) I, global. DOI: 10.1107/S1600536811022653/hb5891sup1.cif

Structure factors: contains datablock(s) I. DOI: 10.1107/S1600536811022653/hb5891Isup2.hkl

Supplementary material file. DOI: 10.1107/S1600536811022653/hb5891Isup3.cdx

Additional supplementary materials:  crystallographic information; 3D view; checkCIF report

## Figures and Tables

**Table 1:** Selected bond lengths (Å)

Sm1—O10	2.435 (3)
Sm1—O5	2.485 (3)
Sm1—O7	2.490 (3)
Sm1—O1	2.495 (3)
Sm1—N2	2.513 (3)
Sm1—O2	2.520 (3)
Sm1—O4	2.528 (3)
Sm1—N4	2.544 (3)
Sm1—O8	2.628 (3)
Sm1—N3	2.661 (3)

**Table 2:** Hydrogen-bond geometry (Å, °)

*D*—H⋯*A*	*D*—H	H⋯*A*	*D*⋯*A*	*D*—H⋯*A*
N1—H1⋯O12	0.86	1.99	2.807 (5)	159
N5—H5⋯O9^i^	0.86	2.10	2.947 (4)	169
N10—H10*A*⋯N11^ii^	0.85 (2)	1.90 (3)	2.727 (6)	166 (6)

Symmetry codes: (i) 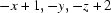; (ii) 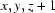.

**Fig. 1.**
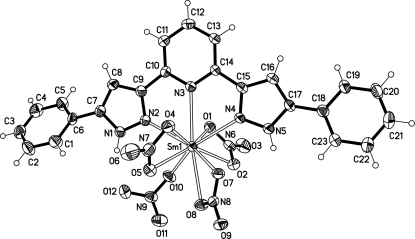
The molecular structure of (I), showing 30% probability displacement ellipsoids.

**Fig. 2.**
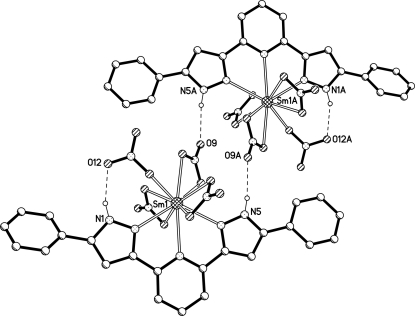
View of a dimer structure constructed through hydrogen bonding interactions in (I). Hydrogen atoms of carbon atoms have been omitted for clarity.

## supplementary crystallographic information

### Comment

Pyrazole derivatives are an important class of organic photochromic compounds. 2,6-Bis-(pyrazolyl)pyridine ligands can be coordinated with transitional metal ions and lanthanide metal ions. Herein we reported the crystal structure of a samarium(III) complex with a bis-(pyrazole) ligand, 2,6-bis-(5-phenyl)-1*H*-pyrazol-3-yl)pyridine (H_2_BPPP).

The atom-numbering scheme of (I) is shown in Fig. 1. The Sm^III^ atom is ten-coordinated by three N atoms from the bis-(pyrazole) ligand and seven O atoms from four nitrate anions. A dimer structure is formed through hydrogen interactions between the oxygen atoms of nitrate anions and the nitrogen atoms of pyrazolyl –NH groups [N5—H5···O9^i^, 2.947 (4) Å, 168.8°, symmetric code i: (-*x* + 1,-*y*,-*z* + 2)].

### Experimental

The bis-(pyrazole) ligand, H_2_BPPP, was prepared according to the literature method (Zhao *et al.*, 2009). H_2_BPPP (0.2 mmol) and Sm(NO_3_)_3_.6H_2_O (0.2 mmol) were dissolved in a DMF (5 ml) and MeOH (5 ml) mixed solvent. Then difuse the solution with ethyl ether. After one week, colourless blocks were obtained. Elmental analysis for C_33_H_28_N_11_O_12_Sm calculated: C 43.03, H 3.06, N 16.73%; found: C 42.81, H 3.08, N1 16.89%.

### Refinement

The H atom of the pyridinium N atom was located in a difference Fourier map and refined with restrained the N—H bond length [0.86 (2) Å] and fixed the isotropic displancement parameter [*U*_iso_(H) = 1.2 *U*_eq_(N)]. The H atoms were placed at calculated positions (C—H = 0.93 Å, N—H = 0.86 Å) and refined as riding with *U*_iso_(H) = 1.2 *U*_eq_(carrier).

### Crystal data

**Table:**

(C_5_H_6_N)[Sm(NO_3_)_4_(C_23_H_17_N_5_)]·C_5_H_5_N	*Z* = 2
*M**_r_* = 921.01	*F*(000) = 922
Triclinic, *P*1	*D*_x_ = 1.656 Mg m^−^^3^
Hall symbol: -P 1	Mo *K*α radiation, λ = 0.71073 Å
*a* = 10.5234 (19) Å	Cell parameters from 3698 reflections
*b* = 12.826 (2) Å	θ = 2.4–24.6°
*c* = 14.190 (3) Å	µ = 1.67 mm^−^^1^
α = 75.970 (2)°	*T* = 296 K
β = 86.453 (2)°	Block, colorless
γ = 84.108 (2)°	0.18 × 0.12 × 0.10 mm
*V* = 1847.0 (6) Å^3^	

### Data collection

**Table:**

Bruker APEXII CCD diffractometer	6391 independent reflections
Radiation source: fine-focus sealed tube	5442 reflections with *I* > 2σ(*I*)
graphite	*R*_int_ = 0.020
φ and ω scans	θ_max_ = 25.1°, θ_min_ = 1.5°
Absorption correction: multi-scan (*SADABS*; Bruker, 2005)	*h* = −12→12
*T*_min_ = 0.753, *T*_max_ = 0.851	*k* = −15→14
9121 measured reflections	*l* = −15→16

### Refinement

**Table:**

Refinement on *F*^2^	Primary atom site location: structure-invariant direct methods
Least-squares matrix: full	Secondary atom site location: difference Fourier map
*R*[*F*^2^ > 2σ(*F*^2^)] = 0.035	Hydrogen site location: inferred from neighbouring sites
*wR*(*F*^2^) = 0.086	H atoms treated by a mixture of independent and constrained refinement
*S* = 1.09	*w* = 1/[σ^2^(*F*_o_^2^) + (0.039*P*)^2^ + 0.1955*P*] where *P* = (*F*_o_^2^ + 2*F*_c_^2^)/3
6391 reflections	(Δ/σ)_max_ = 0.001
517 parameters	Δρ_max_ = 0.95 e Å^−^^3^
1 restraint	Δρ_min_ = −0.67 e Å^−^^3^

### Special details

**Table:**

Geometry. All e.s.d.'s (except the e.s.d. in the dihedral angle between two l.s. planes) are estimated using the full covariance matrix. The cell e.s.d.'s are taken into account individually in the estimation of e.s.d.'s in distances, angles and torsion angles; correlations between e.s.d.'s in cell parameters are only used when they are defined by crystal symmetry. An approximate (isotropic) treatment of cell e.s.d.'s is used for estimating e.s.d.'s involving l.s. planes.
Refinement. Refinement of *F*^2^ against ALL reflections. The weighted *R*-factor *wR* and goodness of fit *S* are based on *F*^2^, conventional *R*-factors *R* are based on *F*, with *F* set to zero for negative *F*^2^. The threshold expression of *F*^2^ > σ(*F*^2^) is used only for calculating *R*-factors(gt) *etc*. and is not relevant to the choice of reflections for refinement. *R*-factors based on *F*^2^ are statistically about twice as large as those based on *F*, and *R*- factors based on ALL data will be even larger.

### Fractional atomic coordinates and isotropic or equivalent isotropic displacement parameters (Å^2^)

**Table:**

	*x*	*y*	*z*	*U*_iso_*/*U*_eq_	
Sm1	0.392775 (19)	0.180975 (17)	0.740894 (14)	0.03930 (9)	
N1	0.2421 (3)	0.4292 (3)	0.6143 (2)	0.0504 (9)	
H1	0.2027	0.4380	0.6672	0.060*	
N2	0.3222 (3)	0.3421 (3)	0.6087 (2)	0.0489 (9)	
N3	0.4891 (3)	0.1922 (3)	0.5612 (2)	0.0422 (8)	
N4	0.5500 (3)	0.0274 (3)	0.7098 (2)	0.0449 (8)	
N5	0.5925 (3)	−0.0668 (3)	0.7709 (2)	0.0478 (9)	
H5	0.5715	−0.0839	0.8318	0.057*	
N6	0.2324 (3)	0.0111 (3)	0.7281 (3)	0.0517 (9)	
N7	0.5444 (4)	0.3545 (3)	0.7586 (3)	0.0619 (11)	
N8	0.4712 (3)	0.1241 (3)	0.9461 (2)	0.0419 (8)	
N9	0.1128 (3)	0.3034 (4)	0.8302 (3)	0.0530 (10)	
O1	0.2555 (3)	0.0884 (3)	0.6556 (2)	0.0523 (8)	
O2	0.2874 (3)	0.0087 (3)	0.8059 (2)	0.0564 (8)	
O3	0.1625 (3)	−0.0575 (3)	0.7225 (3)	0.0785 (11)	
O4	0.5834 (3)	0.2912 (3)	0.7043 (2)	0.0570 (8)	
O5	0.4348 (3)	0.3404 (3)	0.8008 (2)	0.0607 (8)	
O6	0.6086 (5)	0.4237 (4)	0.7694 (3)	0.1002 (14)	
O7	0.5450 (3)	0.1149 (2)	0.87455 (19)	0.0475 (7)	
O8	0.3550 (3)	0.1487 (3)	0.9302 (2)	0.0518 (8)	
O9	0.5139 (3)	0.1087 (3)	1.0279 (2)	0.0550 (8)	
O10	0.1739 (3)	0.2394 (3)	0.7838 (2)	0.0574 (8)	
O11	0.0564 (4)	0.2662 (3)	0.9064 (3)	0.0858 (12)	
O12	0.1077 (5)	0.4011 (3)	0.7945 (3)	0.0980 (14)	
C1	0.1409 (5)	0.6611 (4)	0.5859 (4)	0.0665 (14)	
H1A	0.1880	0.6357	0.6415	0.080*	
C2	0.0655 (5)	0.7596 (5)	0.5734 (4)	0.0762 (16)	
H2	0.0650	0.8009	0.6190	0.091*	
C3	−0.0078 (5)	0.7946 (4)	0.4930 (4)	0.0677 (14)	
H3	−0.0597	0.8591	0.4849	0.081*	
C4	−0.0047 (5)	0.7352 (5)	0.4254 (4)	0.0704 (15)	
H4	−0.0543	0.7594	0.3710	0.084*	
C5	0.0721 (5)	0.6386 (4)	0.4371 (4)	0.0600 (13)	
H5A	0.0733	0.5985	0.3904	0.072*	
C6	0.1461 (4)	0.6014 (3)	0.5168 (3)	0.0463 (10)	
C7	0.2301 (4)	0.5016 (4)	0.5277 (3)	0.0483 (11)	
C8	0.3085 (4)	0.4594 (4)	0.4619 (3)	0.0511 (11)	
H8	0.3216	0.4898	0.3960	0.061*	
C9	0.3643 (4)	0.3606 (4)	0.5159 (3)	0.0466 (10)	
C10	0.4582 (4)	0.2795 (3)	0.4881 (3)	0.0433 (10)	
C11	0.5092 (4)	0.2889 (4)	0.3947 (3)	0.0512 (11)	
H11	0.4845	0.3487	0.3456	0.061*	
C12	0.5964 (5)	0.2093 (4)	0.3751 (3)	0.0577 (12)	
H12A	0.6333	0.2151	0.3130	0.069*	
C13	0.6286 (4)	0.1200 (4)	0.4492 (3)	0.0502 (11)	
H13	0.6868	0.0647	0.4372	0.060*	
C14	0.5737 (4)	0.1134 (3)	0.5414 (3)	0.0394 (9)	
C15	0.6042 (4)	0.0241 (3)	0.6232 (3)	0.0415 (10)	
C16	0.6808 (4)	−0.0740 (4)	0.6299 (3)	0.0473 (11)	
H16	0.7284	−0.0958	0.5795	0.057*	
C17	0.6717 (4)	−0.1313 (4)	0.7253 (3)	0.0436 (10)	
C18	0.7312 (4)	−0.2360 (4)	0.7754 (3)	0.0465 (10)	
C19	0.7853 (4)	−0.3084 (4)	0.7218 (4)	0.0574 (12)	
H19	0.7828	−0.2893	0.6544	0.069*	
C20	0.8418 (5)	−0.4070 (4)	0.7672 (4)	0.0698 (15)	
H20	0.8782	−0.4539	0.7302	0.084*	
C21	0.8455 (5)	−0.4376 (4)	0.8666 (4)	0.0698 (14)	
H21	0.8833	−0.5051	0.8972	0.084*	
C22	0.7923 (5)	−0.3669 (4)	0.9206 (4)	0.0680 (14)	
H22	0.7950	−0.3865	0.9880	0.082*	
C23	0.7357 (4)	−0.2684 (4)	0.8757 (3)	0.0568 (12)	
H23	0.6995	−0.2220	0.9132	0.068*	
N10	0.8105 (4)	0.1433 (5)	0.9215 (5)	0.0802 (15)	
H10A	0.787 (5)	0.197 (3)	0.946 (4)	0.096*	
N11	0.7357 (6)	0.2897 (4)	0.0295 (4)	0.0979 (17)	
C24	0.8310 (6)	0.1677 (5)	0.8235 (6)	0.0891 (19)	
H24	0.8168	0.2386	0.7877	0.107*	
C25	0.8715 (7)	0.0899 (8)	0.7790 (5)	0.108 (3)	
H25	0.8864	0.1058	0.7120	0.130*	
C26	0.8898 (6)	−0.0067 (7)	0.8290 (7)	0.104 (3)	
H26	0.9183	−0.0606	0.7971	0.125*	
C27	0.8699 (6)	−0.0339 (6)	0.9241 (6)	0.092 (2)	
H27	0.8839	−0.1056	0.9579	0.110*	
C28	0.8300 (5)	0.0413 (6)	0.9714 (4)	0.0766 (18)	
H28	0.8157	0.0231	1.0385	0.092*	
C29	0.6193 (6)	0.3324 (5)	0.0444 (6)	0.099 (2)	
H29	0.5524	0.3147	0.0131	0.119*	
C30	0.5930 (6)	0.4013 (5)	0.1035 (5)	0.0885 (18)	
H30	0.5094	0.4299	0.1119	0.106*	
C31	0.6887 (6)	0.4285 (4)	0.1506 (4)	0.0748 (16)	
H31	0.6721	0.4765	0.1905	0.090*	
C32	0.8090 (6)	0.3838 (5)	0.1376 (4)	0.0813 (17)	
H32	0.8770	0.4010	0.1681	0.098*	
C33	0.8288 (6)	0.3127 (5)	0.0788 (5)	0.0898 (19)	
H33	0.9105	0.2790	0.0730	0.108*	

### Atomic displacement parameters (Å^2^)

**Table:**

	*U*^11^	*U*^22^	*U*^33^	*U*^12^	*U*^13^	*U*^23^
Sm1	0.04350 (14)	0.03907 (15)	0.03323 (12)	0.00603 (9)	−0.00094 (9)	−0.00872 (9)
N1	0.062 (2)	0.043 (2)	0.039 (2)	0.0179 (19)	−0.0016 (17)	−0.0065 (17)
N2	0.058 (2)	0.043 (2)	0.0389 (19)	0.0131 (18)	−0.0017 (16)	−0.0050 (17)
N3	0.050 (2)	0.040 (2)	0.0363 (18)	0.0014 (17)	−0.0012 (15)	−0.0111 (16)
N4	0.052 (2)	0.042 (2)	0.0368 (18)	0.0128 (17)	0.0011 (16)	−0.0103 (17)
N5	0.053 (2)	0.049 (2)	0.0371 (19)	0.0123 (18)	0.0016 (16)	−0.0092 (17)
N6	0.047 (2)	0.056 (3)	0.056 (2)	0.004 (2)	0.0077 (19)	−0.027 (2)
N7	0.089 (3)	0.049 (3)	0.046 (2)	−0.014 (2)	−0.007 (2)	−0.005 (2)
N8	0.052 (2)	0.037 (2)	0.0356 (19)	−0.0045 (17)	−0.0018 (16)	−0.0073 (15)
N9	0.047 (2)	0.066 (3)	0.047 (2)	0.005 (2)	−0.0030 (18)	−0.018 (2)
O1	0.0604 (19)	0.052 (2)	0.0443 (17)	0.0069 (16)	−0.0089 (15)	−0.0139 (16)
O2	0.063 (2)	0.056 (2)	0.0466 (18)	−0.0021 (16)	0.0028 (15)	−0.0092 (15)
O3	0.073 (2)	0.078 (3)	0.100 (3)	−0.032 (2)	0.012 (2)	−0.043 (2)
O4	0.065 (2)	0.057 (2)	0.0485 (18)	−0.0115 (17)	0.0061 (15)	−0.0108 (16)
O5	0.074 (2)	0.052 (2)	0.0581 (19)	−0.0052 (18)	0.0071 (17)	−0.0183 (16)
O6	0.141 (4)	0.097 (3)	0.077 (3)	−0.068 (3)	0.006 (3)	−0.030 (2)
O7	0.0469 (16)	0.056 (2)	0.0365 (15)	0.0055 (14)	0.0024 (13)	−0.0105 (14)
O8	0.0436 (17)	0.065 (2)	0.0451 (16)	−0.0027 (15)	0.0029 (13)	−0.0110 (15)
O9	0.066 (2)	0.065 (2)	0.0343 (16)	−0.0032 (17)	−0.0082 (14)	−0.0113 (15)
O10	0.0515 (18)	0.066 (2)	0.0565 (19)	0.0143 (16)	−0.0005 (15)	−0.0265 (17)
O11	0.086 (3)	0.097 (3)	0.072 (2)	−0.009 (2)	0.029 (2)	−0.023 (2)
O12	0.147 (4)	0.051 (3)	0.085 (3)	0.014 (3)	0.032 (3)	−0.015 (2)
C1	0.077 (3)	0.069 (4)	0.053 (3)	0.012 (3)	−0.017 (3)	−0.016 (3)
C2	0.089 (4)	0.062 (4)	0.082 (4)	0.017 (3)	−0.010 (3)	−0.033 (3)
C3	0.066 (3)	0.052 (3)	0.072 (4)	0.015 (3)	0.000 (3)	0.001 (3)
C4	0.061 (3)	0.076 (4)	0.064 (3)	0.013 (3)	−0.018 (3)	0.001 (3)
C5	0.065 (3)	0.056 (3)	0.057 (3)	0.012 (3)	−0.022 (2)	−0.013 (3)
C6	0.052 (3)	0.034 (3)	0.049 (3)	0.005 (2)	−0.011 (2)	−0.003 (2)
C7	0.053 (3)	0.042 (3)	0.046 (2)	0.003 (2)	−0.008 (2)	−0.005 (2)
C8	0.060 (3)	0.050 (3)	0.038 (2)	0.005 (2)	−0.002 (2)	−0.005 (2)
C9	0.048 (2)	0.046 (3)	0.043 (2)	0.005 (2)	−0.0015 (19)	−0.008 (2)
C10	0.051 (2)	0.036 (3)	0.040 (2)	−0.004 (2)	−0.0011 (19)	−0.005 (2)
C11	0.060 (3)	0.047 (3)	0.042 (2)	−0.001 (2)	0.003 (2)	−0.005 (2)
C12	0.067 (3)	0.066 (3)	0.038 (2)	−0.004 (3)	0.008 (2)	−0.011 (2)
C13	0.057 (3)	0.046 (3)	0.045 (2)	0.008 (2)	0.010 (2)	−0.014 (2)
C14	0.043 (2)	0.035 (2)	0.039 (2)	0.0028 (19)	0.0024 (18)	−0.0112 (19)
C15	0.038 (2)	0.045 (3)	0.043 (2)	0.0017 (19)	0.0006 (18)	−0.017 (2)
C16	0.047 (2)	0.050 (3)	0.046 (2)	0.002 (2)	0.0066 (19)	−0.020 (2)
C17	0.042 (2)	0.042 (3)	0.048 (2)	0.005 (2)	−0.0009 (19)	−0.018 (2)
C18	0.041 (2)	0.049 (3)	0.049 (2)	0.004 (2)	−0.0023 (19)	−0.014 (2)
C19	0.063 (3)	0.050 (3)	0.058 (3)	0.014 (2)	−0.003 (2)	−0.016 (2)
C20	0.077 (4)	0.056 (4)	0.078 (4)	0.021 (3)	−0.009 (3)	−0.027 (3)
C21	0.076 (4)	0.045 (3)	0.083 (4)	0.010 (3)	−0.017 (3)	−0.007 (3)
C22	0.085 (4)	0.050 (3)	0.064 (3)	0.009 (3)	−0.008 (3)	−0.007 (3)
C23	0.065 (3)	0.048 (3)	0.055 (3)	0.009 (2)	0.003 (2)	−0.014 (2)
N10	0.046 (2)	0.101 (5)	0.116 (5)	−0.006 (3)	0.005 (3)	−0.071 (4)
N11	0.095 (4)	0.080 (4)	0.138 (5)	−0.007 (3)	0.005 (4)	−0.066 (4)
C24	0.080 (4)	0.076 (5)	0.102 (5)	−0.022 (4)	−0.020 (4)	0.007 (4)
C25	0.112 (6)	0.162 (8)	0.066 (4)	−0.072 (6)	0.025 (4)	−0.041 (5)
C26	0.072 (4)	0.108 (6)	0.152 (8)	−0.039 (4)	0.053 (5)	−0.070 (6)
C27	0.060 (4)	0.074 (5)	0.133 (6)	−0.005 (3)	−0.003 (4)	−0.008 (5)
C28	0.058 (3)	0.114 (6)	0.057 (3)	−0.029 (4)	0.004 (3)	−0.012 (4)
C29	0.086 (5)	0.087 (5)	0.141 (6)	−0.014 (4)	−0.004 (4)	−0.055 (5)
C30	0.074 (4)	0.085 (5)	0.109 (5)	0.002 (3)	0.008 (4)	−0.034 (4)
C31	0.102 (5)	0.054 (4)	0.068 (3)	−0.006 (3)	0.003 (3)	−0.015 (3)
C32	0.086 (4)	0.084 (5)	0.079 (4)	−0.006 (4)	−0.008 (3)	−0.028 (3)
C33	0.081 (4)	0.088 (5)	0.102 (5)	0.014 (4)	0.001 (4)	−0.036 (4)

### Geometric parameters (Å, °)

**Table:**

Sm1—O10	2.435 (3)	C9—C10	1.468 (6)
Sm1—O5	2.485 (3)	C10—C11	1.380 (6)
Sm1—O7	2.490 (3)	C11—C12	1.370 (6)
Sm1—O1	2.495 (3)	C11—H11	0.9300
Sm1—N2	2.513 (3)	C12—C13	1.383 (6)
Sm1—O2	2.520 (3)	C12—H12A	0.9300
Sm1—O4	2.528 (3)	C13—C14	1.383 (5)
Sm1—N4	2.544 (3)	C13—H13	0.9300
Sm1—O8	2.628 (3)	C14—C15	1.445 (6)
Sm1—N3	2.661 (3)	C15—C16	1.409 (6)
N1—N2	1.344 (4)	C16—C17	1.377 (6)
N1—C7	1.353 (5)	C16—H16	0.9300
N1—H1	0.8600	C17—C18	1.456 (6)
N2—C9	1.336 (5)	C18—C23	1.385 (6)
N3—C14	1.347 (5)	C18—C19	1.394 (6)
N3—C10	1.357 (5)	C19—C20	1.366 (6)
N4—C15	1.330 (5)	C19—H19	0.9300
N4—N5	1.356 (5)	C20—C21	1.372 (7)
N5—C17	1.360 (5)	C20—H20	0.9300
N5—H5	0.8600	C21—C22	1.379 (7)
N6—O3	1.223 (5)	C21—H21	0.9300
N6—O2	1.271 (4)	C22—C23	1.363 (6)
N6—O1	1.275 (5)	C22—H22	0.9300
N7—O6	1.212 (5)	C23—H23	0.9300
N7—O4	1.271 (5)	N10—C28	1.330 (8)
N7—O5	1.276 (5)	N10—C24	1.358 (8)
N8—O9	1.234 (4)	N10—H10A	0.85 (2)
N8—O8	1.251 (4)	N11—C29	1.316 (7)
N8—O7	1.261 (4)	N11—C33	1.334 (7)
N9—O11	1.215 (5)	C24—C25	1.327 (9)
N9—O12	1.229 (5)	C24—H24	0.9300
N9—O10	1.273 (4)	C25—C26	1.272 (10)
C1—C6	1.379 (6)	C25—H25	0.9300
C1—C2	1.400 (7)	C26—C27	1.319 (10)
C1—H1A	0.9300	C26—H26	0.9300
C2—C3	1.373 (7)	C27—C28	1.323 (8)
C2—H2	0.9300	C27—H27	0.9300
C3—C4	1.360 (7)	C28—H28	0.9300
C3—H3	0.9300	C29—C30	1.357 (8)
C4—C5	1.388 (7)	C29—H29	0.9300
C4—H4	0.9300	C30—C31	1.360 (8)
C5—C6	1.373 (6)	C30—H30	0.9300
C5—H5A	0.9300	C31—C32	1.356 (8)
C6—C7	1.460 (6)	C31—H31	0.9300
C7—C8	1.380 (6)	C32—C33	1.371 (7)
C8—C9	1.403 (6)	C32—H32	0.9300
C8—H8	0.9300	C33—H33	0.9300
			
O10—Sm1—O5	81.13 (11)	C6—C5—C4	120.7 (5)
O10—Sm1—O7	117.91 (9)	C6—C5—H5A	119.6
O5—Sm1—O7	73.20 (10)	C4—C5—H5A	119.6
O10—Sm1—O1	74.81 (10)	C5—C6—C1	118.6 (4)
O5—Sm1—O1	150.70 (11)	C5—C6—C7	120.9 (4)
O7—Sm1—O1	133.29 (10)	C1—C6—C7	120.5 (4)
O10—Sm1—N2	73.12 (11)	N1—C7—C8	106.2 (4)
O5—Sm1—N2	74.73 (11)	N1—C7—C6	121.8 (4)
O7—Sm1—N2	143.51 (11)	C8—C7—C6	132.0 (4)
O1—Sm1—N2	82.38 (11)	C7—C8—C9	105.2 (4)
O10—Sm1—O2	75.23 (11)	C7—C8—H8	127.4
O5—Sm1—O2	137.11 (10)	C9—C8—H8	127.4
O7—Sm1—O2	86.95 (10)	N2—C9—C8	110.9 (4)
O1—Sm1—O2	51.04 (10)	N2—C9—C10	117.5 (4)
N2—Sm1—O2	128.99 (11)	C8—C9—C10	131.5 (4)
O10—Sm1—O4	128.02 (11)	N3—C10—C11	122.1 (4)
O5—Sm1—O4	51.07 (10)	N3—C10—C9	114.6 (4)
O7—Sm1—O4	71.20 (10)	C11—C10—C9	123.3 (4)
O1—Sm1—O4	139.13 (10)	C12—C11—C10	119.4 (4)
N2—Sm1—O4	75.30 (11)	C12—C11—H11	120.3
O2—Sm1—O4	153.47 (11)	C10—C11—H11	120.3
O10—Sm1—N4	147.20 (11)	C11—C12—C13	118.9 (4)
O5—Sm1—N4	128.81 (11)	C11—C12—H12A	120.6
O7—Sm1—N4	68.22 (10)	C13—C12—H12A	120.6
O1—Sm1—N4	79.16 (10)	C14—C13—C12	119.7 (4)
N2—Sm1—N4	122.84 (11)	C14—C13—H13	120.2
O2—Sm1—N4	72.95 (11)	C12—C13—H13	120.2
O4—Sm1—N4	84.77 (11)	N3—C14—C13	121.6 (4)
O10—Sm1—O8	68.59 (10)	N3—C14—C15	115.3 (3)
O5—Sm1—O8	67.89 (10)	C13—C14—C15	123.1 (4)
O7—Sm1—O8	49.48 (9)	N4—C15—C16	109.8 (4)
O1—Sm1—O8	116.62 (10)	N4—C15—C14	118.5 (4)
N2—Sm1—O8	129.41 (10)	C16—C15—C14	131.6 (4)
O2—Sm1—O8	70.29 (10)	C17—C16—C15	106.9 (4)
O4—Sm1—O8	103.96 (9)	C17—C16—H16	126.5
N4—Sm1—O8	107.13 (10)	C15—C16—H16	126.5
O10—Sm1—N3	125.62 (10)	N5—C17—C16	105.0 (4)
O5—Sm1—N3	111.82 (10)	N5—C17—C18	123.2 (4)
O7—Sm1—N3	116.36 (9)	C16—C17—C18	131.8 (4)
O1—Sm1—N3	71.09 (10)	C23—C18—C19	117.5 (4)
N2—Sm1—N3	61.55 (11)	C23—C18—C17	122.7 (4)
O2—Sm1—N3	111.06 (10)	C19—C18—C17	119.8 (4)
O4—Sm1—N3	68.21 (10)	C20—C19—C18	120.8 (5)
N4—Sm1—N3	61.30 (10)	C20—C19—H19	119.6
O8—Sm1—N3	165.78 (10)	C18—C19—H19	119.6
N2—N1—C7	112.7 (3)	C19—C20—C21	120.8 (5)
N2—N1—H1	123.7	C19—C20—H20	119.6
C7—N1—H1	123.7	C21—C20—H20	119.6
C9—N2—N1	105.0 (3)	C20—C21—C22	119.0 (5)
C9—N2—Sm1	125.5 (3)	C20—C21—H21	120.5
N1—N2—Sm1	129.5 (2)	C22—C21—H21	120.5
C14—N3—C10	118.3 (3)	C23—C22—C21	120.4 (5)
C14—N3—Sm1	120.9 (3)	C23—C22—H22	119.8
C10—N3—Sm1	120.8 (3)	C21—C22—H22	119.8
C15—N4—N5	105.5 (3)	C22—C23—C18	121.4 (4)
C15—N4—Sm1	124.1 (3)	C22—C23—H23	119.3
N5—N4—Sm1	130.3 (2)	C18—C23—H23	119.3
N4—N5—C17	112.7 (3)	C28—N10—C24	119.4 (5)
N4—N5—H5	123.6	C28—N10—H10A	125 (4)
C17—N5—H5	123.6	C24—N10—H10A	115 (4)
O3—N6—O2	122.0 (4)	C29—N11—C33	117.3 (5)
O3—N6—O1	121.8 (4)	C25—C24—N10	119.7 (7)
O2—N6—O1	116.1 (4)	C25—C24—H24	120.2
O6—N7—O4	121.5 (5)	N10—C24—H24	120.2
O6—N7—O5	122.4 (4)	C26—C25—C24	119.3 (7)
O4—N7—O5	116.1 (4)	C26—C25—H25	120.3
O9—N8—O8	122.4 (3)	C24—C25—H25	120.3
O9—N8—O7	120.2 (3)	C25—C26—C27	122.8 (7)
O8—N8—O7	117.3 (3)	C25—C26—H26	118.6
O11—N9—O12	121.7 (4)	C27—C26—H26	118.6
O11—N9—O10	119.3 (4)	C26—C27—C28	119.8 (7)
O12—N9—O10	119.0 (4)	C26—C27—H27	120.1
N6—O1—Sm1	96.9 (2)	C28—C27—H27	120.1
N6—O2—Sm1	95.8 (2)	C27—C28—N10	119.0 (6)
N7—O4—Sm1	95.4 (3)	C27—C28—H28	120.5
N7—O5—Sm1	97.3 (2)	N10—C28—H28	120.5
N8—O7—Sm1	99.6 (2)	N11—C29—C30	122.7 (6)
N8—O8—Sm1	93.1 (2)	N11—C29—H29	118.7
N9—O10—Sm1	140.0 (3)	C30—C29—H29	118.7
C6—C1—C2	120.7 (5)	C29—C30—C31	120.1 (6)
C6—C1—H1A	119.6	C29—C30—H30	120.0
C2—C1—H1A	119.6	C31—C30—H30	120.0
C3—C2—C1	119.3 (5)	C32—C31—C30	118.2 (6)
C3—C2—H2	120.3	C32—C31—H31	120.9
C1—C2—H2	120.3	C30—C31—H31	120.9
C4—C3—C2	120.2 (5)	C31—C32—C33	118.9 (6)
C4—C3—H3	119.9	C31—C32—H32	120.6
C2—C3—H3	119.9	C33—C32—H32	120.6
C3—C4—C5	120.4 (5)	N11—C33—C32	122.8 (6)
C3—C4—H4	119.8	N11—C33—H33	118.6
C5—C4—H4	119.8	C32—C33—H33	118.6
			
C7—N1—N2—C9	1.1 (5)	N2—Sm1—O7—N8	100.4 (3)
C7—N1—N2—Sm1	178.0 (3)	O2—Sm1—O7—N8	−70.6 (2)
O10—Sm1—N2—C9	−150.8 (4)	O4—Sm1—O7—N8	124.7 (2)
O5—Sm1—N2—C9	124.0 (4)	N4—Sm1—O7—N8	−143.4 (2)
O7—Sm1—N2—C9	94.8 (4)	O8—Sm1—O7—N8	−4.0 (2)
O1—Sm1—N2—C9	−74.5 (3)	N3—Sm1—O7—N8	177.4 (2)
O2—Sm1—N2—C9	−96.9 (4)	O9—N8—O8—Sm1	173.4 (3)
O4—Sm1—N2—C9	71.0 (3)	O7—N8—O8—Sm1	−6.8 (3)
N4—Sm1—N2—C9	−2.7 (4)	O10—Sm1—O8—N8	−171.2 (2)
O8—Sm1—N2—C9	167.2 (3)	O5—Sm1—O8—N8	−82.4 (2)
N3—Sm1—N2—C9	−1.9 (3)	O7—Sm1—O8—N8	4.0 (2)
O10—Sm1—N2—N1	32.9 (3)	O1—Sm1—O8—N8	129.5 (2)
O5—Sm1—N2—N1	−52.3 (3)	N2—Sm1—O8—N8	−127.8 (2)
O7—Sm1—N2—N1	−81.5 (4)	O2—Sm1—O8—N8	107.4 (2)
O1—Sm1—N2—N1	109.2 (3)	O4—Sm1—O8—N8	−45.5 (2)
O2—Sm1—N2—N1	86.8 (4)	N4—Sm1—O8—N8	43.3 (2)
O4—Sm1—N2—N1	−105.3 (4)	N3—Sm1—O8—N8	9.3 (5)
N4—Sm1—N2—N1	−179.0 (3)	O11—N9—O10—Sm1	−110.3 (5)
O8—Sm1—N2—N1	−9.1 (4)	O12—N9—O10—Sm1	72.9 (6)
N3—Sm1—N2—N1	−178.2 (4)	O5—Sm1—O10—N9	−12.6 (4)
O10—Sm1—N3—C14	−142.6 (3)	O7—Sm1—O10—N9	53.0 (5)
O5—Sm1—N3—C14	122.7 (3)	O1—Sm1—O10—N9	−175.7 (5)
O7—Sm1—N3—C14	41.2 (3)	N2—Sm1—O10—N9	−89.2 (5)
O1—Sm1—N3—C14	−88.5 (3)	O2—Sm1—O10—N9	131.3 (5)
N2—Sm1—N3—C14	−180.0 (3)	O4—Sm1—O10—N9	−34.2 (5)
O2—Sm1—N3—C14	−56.1 (3)	N4—Sm1—O10—N9	145.7 (4)
O4—Sm1—N3—C14	95.4 (3)	O8—Sm1—O10—N9	57.1 (4)
N4—Sm1—N3—C14	−0.7 (3)	N3—Sm1—O10—N9	−123.1 (4)
O8—Sm1—N3—C14	36.7 (5)	C6—C1—C2—C3	2.6 (8)
O10—Sm1—N3—C10	40.1 (3)	C1—C2—C3—C4	−1.5 (8)
O5—Sm1—N3—C10	−54.6 (3)	C2—C3—C4—C5	0.3 (8)
O7—Sm1—N3—C10	−136.0 (3)	C3—C4—C5—C6	−0.1 (8)
O1—Sm1—N3—C10	94.2 (3)	C4—C5—C6—C1	1.1 (7)
N2—Sm1—N3—C10	2.7 (3)	C4—C5—C6—C7	−178.0 (4)
O2—Sm1—N3—C10	126.7 (3)	C2—C1—C6—C5	−2.4 (7)
O4—Sm1—N3—C10	−81.9 (3)	C2—C1—C6—C7	176.7 (5)
N4—Sm1—N3—C10	−178.0 (3)	N2—N1—C7—C8	−0.7 (5)
O8—Sm1—N3—C10	−140.5 (4)	N2—N1—C7—C6	179.0 (4)
O10—Sm1—N4—C15	112.1 (3)	C5—C6—C7—N1	−138.8 (5)
O5—Sm1—N4—C15	−96.0 (3)	C1—C6—C7—N1	42.1 (6)
O7—Sm1—N4—C15	−139.9 (3)	C5—C6—C7—C8	40.8 (7)
O1—Sm1—N4—C15	74.2 (3)	C1—C6—C7—C8	−138.3 (5)
N2—Sm1—N4—C15	0.7 (4)	N1—C7—C8—C9	0.0 (5)
O2—Sm1—N4—C15	126.6 (3)	C6—C7—C8—C9	−179.6 (4)
O4—Sm1—N4—C15	−68.0 (3)	N1—N2—C9—C8	−1.1 (5)
O8—Sm1—N4—C15	−171.1 (3)	Sm1—N2—C9—C8	−178.1 (3)
N3—Sm1—N4—C15	−0.1 (3)	N1—N2—C9—C10	178.1 (4)
O10—Sm1—N4—N5	−64.8 (4)	Sm1—N2—C9—C10	1.0 (5)
O5—Sm1—N4—N5	87.2 (4)	C7—C8—C9—N2	0.7 (5)
O7—Sm1—N4—N5	43.3 (3)	C7—C8—C9—C10	−178.3 (4)
O1—Sm1—N4—N5	−102.6 (3)	C14—N3—C10—C11	1.0 (6)
N2—Sm1—N4—N5	−176.1 (3)	Sm1—N3—C10—C11	178.3 (3)
O2—Sm1—N4—N5	−50.3 (3)	C14—N3—C10—C9	179.3 (4)
O4—Sm1—N4—N5	115.1 (3)	Sm1—N3—C10—C9	−3.3 (5)
O8—Sm1—N4—N5	12.1 (4)	N2—C9—C10—N3	1.6 (6)
N3—Sm1—N4—N5	−176.9 (4)	C8—C9—C10—N3	−179.4 (4)
C15—N4—N5—C17	−0.7 (5)	N2—C9—C10—C11	180.0 (4)
Sm1—N4—N5—C17	176.6 (3)	C8—C9—C10—C11	−1.1 (7)
O3—N6—O1—Sm1	177.9 (3)	N3—C10—C11—C12	−1.8 (7)
O2—N6—O1—Sm1	−2.9 (3)	C9—C10—C11—C12	180.0 (4)
O10—Sm1—O1—N6	−81.4 (2)	C10—C11—C12—C13	1.6 (7)
O5—Sm1—O1—N6	−117.3 (3)	C11—C12—C13—C14	−0.8 (7)
O7—Sm1—O1—N6	32.9 (3)	C10—N3—C14—C13	0.0 (6)
N2—Sm1—O1—N6	−155.9 (2)	Sm1—N3—C14—C13	−177.4 (3)
O2—Sm1—O1—N6	1.7 (2)	C10—N3—C14—C15	178.7 (4)
O4—Sm1—O1—N6	147.2 (2)	Sm1—N3—C14—C15	1.4 (4)
N4—Sm1—O1—N6	78.5 (2)	C12—C13—C14—N3	0.0 (6)
O8—Sm1—O1—N6	−25.4 (2)	C12—C13—C14—C15	−178.7 (4)
N3—Sm1—O1—N6	141.6 (2)	N5—N4—C15—C16	0.5 (4)
O3—N6—O2—Sm1	−177.9 (3)	Sm1—N4—C15—C16	−177.0 (2)
O1—N6—O2—Sm1	2.9 (3)	N5—N4—C15—C14	178.2 (3)
O10—Sm1—O2—N6	80.5 (2)	Sm1—N4—C15—C14	0.8 (5)
O5—Sm1—O2—N6	139.3 (2)	N3—C14—C15—N4	−1.4 (5)
O7—Sm1—O2—N6	−159.6 (2)	C13—C14—C15—N4	177.3 (4)
O1—Sm1—O2—N6	−1.7 (2)	N3—C14—C15—C16	175.8 (4)
N2—Sm1—O2—N6	27.4 (3)	C13—C14—C15—C16	−5.5 (7)
O4—Sm1—O2—N6	−125.6 (3)	N4—C15—C16—C17	−0.2 (5)
N4—Sm1—O2—N6	−91.4 (2)	C14—C15—C16—C17	−177.5 (4)
O8—Sm1—O2—N6	152.7 (2)	N4—N5—C17—C16	0.6 (5)
N3—Sm1—O2—N6	−42.4 (2)	N4—N5—C17—C18	179.2 (4)
O6—N7—O4—Sm1	177.0 (4)	C15—C16—C17—N5	−0.2 (4)
O5—N7—O4—Sm1	−2.7 (4)	C15—C16—C17—C18	−178.6 (4)
O10—Sm1—O4—N7	29.5 (3)	N5—C17—C18—C23	−14.9 (7)
O5—Sm1—O4—N7	1.6 (2)	C16—C17—C18—C23	163.3 (5)
O7—Sm1—O4—N7	−81.7 (2)	N5—C17—C18—C19	164.5 (4)
O1—Sm1—O4—N7	142.8 (2)	C16—C17—C18—C19	−17.3 (7)
N2—Sm1—O4—N7	83.6 (3)	C23—C18—C19—C20	−0.7 (7)
O2—Sm1—O4—N7	−117.8 (3)	C17—C18—C19—C20	179.9 (4)
N4—Sm1—O4—N7	−150.4 (3)	C18—C19—C20—C21	0.7 (8)
O8—Sm1—O4—N7	−44.0 (3)	C19—C20—C21—C22	−0.6 (8)
N3—Sm1—O4—N7	148.5 (3)	C20—C21—C22—C23	0.6 (8)
O6—N7—O5—Sm1	−177.0 (4)	C21—C22—C23—C18	−0.7 (8)
O4—N7—O5—Sm1	2.7 (4)	C19—C18—C23—C22	0.7 (7)
O10—Sm1—O5—N7	−159.6 (3)	C17—C18—C23—C22	−179.9 (4)
O7—Sm1—O5—N7	77.5 (3)	C28—N10—C24—C25	−0.8 (9)
O1—Sm1—O5—N7	−124.7 (3)	N10—C24—C25—C26	0.4 (10)
N2—Sm1—O5—N7	−84.8 (3)	C24—C25—C26—C27	0.1 (12)
O2—Sm1—O5—N7	143.5 (2)	C25—C26—C27—C28	−0.4 (11)
O4—Sm1—O5—N7	−1.6 (2)	C26—C27—C28—N10	0.1 (9)
N4—Sm1—O5—N7	35.3 (3)	C24—N10—C28—C27	0.5 (8)
O8—Sm1—O5—N7	129.9 (3)	C33—N11—C29—C30	3.0 (11)
N3—Sm1—O5—N7	−34.7 (3)	N11—C29—C30—C31	−0.2 (11)
O9—N8—O7—Sm1	−172.9 (3)	C29—C30—C31—C32	−1.0 (10)
O8—N8—O7—Sm1	7.3 (4)	C30—C31—C32—C33	−0.6 (9)
O10—Sm1—O7—N8	1.0 (3)	C29—N11—C33—C32	−4.7 (11)
O5—Sm1—O7—N8	70.9 (2)	C31—C32—C33—N11	3.6 (10)
O1—Sm1—O7—N8	−94.3 (2)		

### Hydrogen-bond geometry (Å, °)

**Table:**

*D*—H···*A*	*D*—H	H···*A*	*D*···*A*	*D*—H···*A*
N1—H1···O12	0.86	1.99	2.807 (5)	159
N5—H5···O9^i^	0.86	2.10	2.947 (4)	169
N10—H10A···N11^ii^	0.85 (2)	1.90 (3)	2.727 (6)	166 (6)

Symmetry codes: (i) −*x*+1, −*y*, −*z*+2; (ii) *x*, *y*, *z*+1.
